# Supplementary material for: Population pharmacokinetic modelling of primaquine exposures in lactating women and breastfed infants
Source: Nat Commun. 2024 May 8;15:3851. doi: 10.1038/s41467-024-47908-y (PMC11078975; doi:10.1038/s41467-024-47908-y)
Supplement: Supplementary file 1 — Supplementary Information [file 41467_2024_47908_MOESM1_ESM.pdf]

Supplementary material

Population pharmacokinetic modelling of primaquine exposures in lactating women and breastfed infants

Thanaporn Wattanakul, Mary Ellen Gilder, Rose McGready, Warunee Hanpithakpong, Nicholas P. J. Day, Nicholas J. White, François Nosten, Joel Tarning, Richard M. Hoglund

Supplementary Methods

Sensitivity analysis of feeding pattern

The sensitivity analysis was performed to investigate the impact of different feeding frequencies and feeding volumes on the predicted exposure to primaquine and carboxyprimaquine in infants. Feeding volume was calculated using the infant body weight (150 ml/kg/day, body weight 2 to 17 kg) divided by the number of feeds per day. The breastfeeding parameters used for simulations are summarized in the following table;

No. of feed/day	Average volume/feed (ml)	Feeding window (min)	Break (h)	Feeding cycle length (h)
10[a]	140 (30.0-257)	24	2	2.4
8	175 (37.5-321)	30	2.5	3
6	234 (50.0-428)	30	3.5	4
4	351 (75.0-641)	30	5.5	6
2	701 (150-1283)	30	11.5	12

[a] used in the developed mother-to-infant model

The models with varied number of feeds per day were implemented to obtain pharmacokinetic parameter estimates. The final parameter estimates of each model was then used to simulate (n = 1000) the predicted concentration-time profiles and exposure parameters in infants weighing 2 to 17 kg (age 0 to 24 months). The mother's body weight was assumed to be 60 kg.

Sensitivity analysis of MAO-A enzyme maturation

The maturation effect of the monoamine oxidase A (MAO-A) enzyme in infants, responsible for converting primaquine to carboxyprimaquine was incorporated by multiplying the maturation effect (MF) to the apparent clearance of primaquine in infants. In the mother-to-infant model, it was assumed that the enzyme activity at full-term birth was 55% compared to adults. In the sensitivity analysis, the TM_{50} was varied to get different enzyme activity at birth. The TM_{50} implemented in the sensitivity analysis and the derived enzyme activity at full-term birth are presented in the following table;

TM_{50} (months)	Enzyme activity at birth (%)	Age at which enzyme activity reach 90% (years)
7.6[a]	55	4.64
27.6	25	18.9
64.4	12.5	45.0

[a] used in the developed mother-to-infant model. TM_{50} is the PMA at which the clearance in infants is 50% of the mature clearance.

NONMEM code

```
$PROBLEM    The mother-to-infant model of primaquine and carboxyprimaquine

$INPUT
ID                                   ; PARTICIPANT ID
TIME                                 ; TIME (H)
TAD                                  ; TIME AFTER DOSE (H)
AMT                                  ; DOSE AMOUNT OF PRIMAQUINE (NMOL)
EVID                                 ; EVENT ID RECORD
CMT                                  ; COMPARTMENT
LNDV=DV                              ; DEPENDENT VARIABLE (OBSERVED DRUG CONCENTRATION, NMOL/L)
MDV                                  ; MISSING DEPENDENT VARIABLE
OCC                                  ; BLOOD SAMPLING OCCASION
WT                                   ; MOTHER BODY WEIGHT (KG)
INFWT                                ; INFANT BODY WEIGHT (KG)
INFAGE                               ; INFANT AGE (MONTHS)

$DATA
PRQ_Breastmilk_data.csv IGNORE= @

$SUBROUTINE
ADVAN13 TOL=6                        ; SPECIFY SUBROUTINE

$MODEL
COMP= (1)                            ; DOSE COMPARTMENT
COMP= (2)                            ; CENTRAL COMPARTMENT PQ
COMP= (3)                            ; CENTRAL COMPARTMENT CPQ
COMP= (4)                            ; TRANSIT COMPARTMENT 1
COMP= (5)                            ; TRANSIT COMPARTMENT 2
COMP= (6)                            ; TRANSIT COMPARTMENT 3
COMP= (7)                            ; TRANSIT COMPARTMENT 4
COMP= (8)                            ; BREAST MILK COMPARTMENT PQ
COMP= (9)                            ; BREAST MILK COMPARTMENT CPQ
COMP= (10)                           ; INFANT-PQ-DOSE COMPARTMENT
COMP= (11)                           ; INFANT-TRANSIT COMPARTMENT 1 FOR PQ
COMP= (12)                           ; INFANT-TRANSIT COMPARTMENT 2 FOR PQ
COMP= (13)                           ; INFANT-CPQ-DOSE COMPARTMENT
COMP= (14)                           ; INFANT-TRANSIT COMPARTMENT 1 FOR CPQ
COMP= (15)                           ; INFANT-TRANSIT COMPARTMENT 2 FOR CPQ
COMP= (16)                           ; INFANT CENTRAL COMPARTMENT PQ
COMP= (17)                           ; INFANT CENTRAL COMPARTMENT CPQ

$PK
; BETWEEN OCCASION VARIABILITY
OCC1=0
OCC2=0
OCC3=0
OCC4=0

IF (OCC.EQ.1)   OCC1=1
IF (OCC.EQ.2)   OCC2=1
IF (OCC.EQ.3)   OCC3=1
IF (OCC.EQ.4)   OCC4=1

IOVF1= OCC1*ETA (11) + OCC2*ETA (12) +            ; INTER-OCCASION VARIABILITY ON BIOAVAILABILITY
       OCC3*ETA (13) + OCC4*ETA (14)
IOVMTT= OCC1*ETA (15) + OCC2*ETA (16) +           ; INTER-OCCASION VARIABILITY ON MEAN TRANSIT ABSORPTION TIME
        OCC3*ETA (17) + OCC4*ETA (18)

; PK-MODEL
TVF1= THETA (1)                      ; TYPICAL VALUE OF RELATIVE BIOAVAILABILITY
F1= TVF1*EXP (ETA (1) + IOVF1)       ; INDIVIDUAL RELATIVE BIOAVAILABILITY

TVCLP= THETA (2) *(WT/51) **0.75     ; TYPICAL VALUE OF PQ ELIMINATION CLEARANCE
CLP= TVCLP*EXP (ETA (2))             ; INDIVIDUAL PQ ELIMINATION CLEARANCE

TVV2= THETA (3) *(WT/51)             ; TYPICAL VALUE OF PQ CENTRAL VOLUME OF DISTRIBUTION
V2= TVV2*EXP (ETA (3))               ; INDIVIDUAL PQ CENTRAL VOLUME OF DISTRIBUTION


TVCLM= THETA (4) *(WT/51) **0.75     ; TYPICAL VALUE OF CPQ ELIMINATION CLEARANCE
CLM= TVCLM*EXP (ETA (4))             ; INDIVIDUAL CPQ ELIMINATION CLEARANCE
```

```
TVV3= THETA (5) *(WT/51)                    ; TYPICAL VALUE OF CPQ CENTRAL VOLUME OF DISTRIBUTION
V3= TVV3*EXP (ETA (5))                      ; INDIVIDUAL CPQ CENTRAL VOLUME OF DISTRIBUTION

TVMTT= THETA (6)                            ; TYPICAL VALUE OF MEAN TRANSIT ABSORPTION TIME
MTT= TVMTT *EXP (ETA (6) +IOVMTT)           ; INDIVIDUAL MEAN TRANSIT ABSORPTION TIME
NN= 4                                       ; NUMBER OF TRANSIT COMPARTMENTS
KTR= (NN+1)/MTT                             ; TRANSIT COMPARTMENT RATE CONSTANT

TVFM= THETA (7);                            ; TYPICAL VALUE OF FRACTION OF PQ CONVERTED TO CPQ VIA FIRST-PASS METABOLISM
FM= TVFM*EXP (ETA (7));                     ; INDIVIDUAL FRACTION OF PQ CONVERTED TO CPQ VIA FIRST-PASS METABOLISM
LTF= LOG (TVFM / (1-TVFM))                  ; LOGIT TRANSFORMATION OF FM
FM= EXP (LTF+ETA (7))/ (1+EXP (LTF+ETA (7)))

TVQPQ= THETA (8)                            ; TYPICAL VALUE OF INTERCOMPARTMENTAL CLEARANCE BETWEEN CENTRAL AND BREAST MILK
                                            COMPARTMENT OF PQ
QPQ= TVQPQ *EXP (ETA (8))                   ; INDIVIDAUL INTERCOMPARTMENTAL CLEARANCE BETWEEN CENTRAL AND BREAST MILK
                                            COMPARTMENT OF PQ
QCPQ = QPQ                                  ; INDIVIDAUL INTERCOMPARTMENTAL CLEARANCE BETWEEN CENTRAL AND BREAST MILK
                                            COMPARTMENT OF CPQ

FEEDNO= 10                                  ; NUMBER OF FEEDING PER DAY
VMPQ= (0.15*INFWT)/FEEDNO                   ; INDIVIDAUL PQ VOLUME OF DISTRIBUTION OF BREAST MILK COMPARTMENT
VMCPQ= (0.15*INFWT)/FEEDNO                  ; INDIVIDAUL CPQ VOLUME OF DISTRIBUTION OF BREAST MILK COMPARTMENT

TVPC1= THETA (9)                            ; TYPICAL VALUE OF FRACTION OF PQ PLASMA DISTRIBUTED TO BREAST MILK
PC1= TVPC1*EXP (ETA (9))                    ; INDIVIDUAL VALUE OF FRACTION OF PQ PLASMA DISTRIBUTED TO BREAST MILK

TVPC2= THETA (10)                           ; TYPICAL VALUE OF FRACTION OF CPQ PLASMA DISTRIBUTED TO BREAST MILK
PC2= TVPC2*EXP (ETA (10))                   ; INDIVIDUAL VALUE OF FRACTION OF CPQ PLASMA DISTRIBUTED TO BREAST MILK

CFPQ = THETA (11)                           ; CONVERSION FACTOR BETWEEN VENOUS AND CAPPILLARY CONCENTRATION OF PQ
CFCPQ= THETA (12)                           ; CONVERSION FACTOR BETWEEN VENOUS AND CAPPILLARY CONCENTRATION OF CPQ

; INFANT PK PARAMETERS
INFF1= 1                                    ; INFANT RELATIVE BIOAVAILABILITY
TM50= 7.6                                   ; MT50=7.6 MONTHS TO MATCHED MF=0.55 AT BIRTH
MF=(INFAGE+9.2)/((TM50) +( INFAGE+9.2))     ; MATURATION EFFECT ASSUMING FULL-TERM GESTATION AT 9.2 MONTHS
INFCLP= CLP*((INFWT/WT) **0.75) *MF         ; INFANT PQ ELIMINATION CLEARANCE
INFV2= V2*(INFWT/ WT)                       ; INFANT PQ CENTRAL VOLUME OF DISTRIBUTION
INFCLM= CLM*((INFWT/ WT) **0.75)            ; INFANT CPQ ELIMINATION CLEARANCE
INFV3= V3*(INFWT/ WT)                       ; INFANT CPQ CENTRAL VOLUME OF DISTRIBUTION
INFMTT= 0.706                               ; INFANT MEAN TRANSIT ABSORPTION TIME
INFNN= 2                                    ; INFANT NUMBER OF TRANSIT COMPARTMENT
INFKTR= (INFNN+1)/INFMTT                    ; INFANT TRANSIT COMPARTMENT RATE CONSTANT
MTINFPQ= 100                                ; TRANSFER RATE OF CQ FROM BREAST MILK TO INFANT DOSE COMPARTMENT
MTINFCPQ= 100                               ; TRANSFER RATE OF CPQ FROM BREAST MILK TO INFANT DOSE COMPARTMENT

; RATE CONSTANTS
K14= KTR                                    ; RATE CONSTANT
K45= KTR                                    ; RATE CONSTANT
K56= KTR                                    ; RATE CONSTANT
K67= KTR                                    ; RATE CONSTANT
K72= KTR*(1-FM)                             ; RATE CONSTANT
K73= KTR*FM                                 ; RATE CONSTANT
K23= CLP/V2                                 ; RATE CONSTANT
K30= CLM/V3                                 ; RATE CONSTANT
K28= (QPQ/V2) *PC1                          ; RATE CONSTANT
K82= QPQ/VMPQ                               ; RATE CONSTANT
K39= (QCPQ/V3) *PC2                         ; RATE CONSTANT
K93= QCPQ/VMCPQ                             ; RATE CONSTANT
K8T10= MTINFPQ                              ; RATE CONSTANT
K9T13= MTINFCPQ                             ; RATE CONSTANT
K10T11= INFKTR                              ; RATE CONSTANT
K11T12= INFKTR                              ; RATE CONSTANT
K12T16= INFKTR                              ; RATE CONSTANT
K13T14= INFKTR                              ; RATE CONSTANT
K14T15= INFKTR                              ; RATE CONSTANT
K15T17= INFKTR                              ; RATE CONSTANT
K16T17= INFCLP/INFV2                        ; RATE CONSTANT
K17T0= INFCLM/INFV3                         ; RATE CONSTANT

; SCALING FACTORS
S2= V2                                      ;SCALING FACTOR (DOSE = NMOL, CONC= NMOL/L)
```

```
S3= V3                                                      ;SCALING FACTOR (DOSE = NMOL, CONC= NMOL/L)
S8= VMPRQ                                                   ;SCALING FACTOR (DOSE = NMOL, CONC= NMOL/L)
S9= VMCPRQ                                                  ;SCALING FACTOR (DOSE = NMOL, CONC= NMOL/L)
S16= INFV2                                                  ;SCALING FACTOR (DOSE = NMOL, CONC= NMOL/L)
S17= INFV3                                                  ;SCALING FACTOR (DOSE = NMOL, CONC= NMOL/L)

$DES
; SQUARE-WAVE FUNCTIONS                                     ; SQUARE-WAVE FUNCTIONS
PI= 3.14159265359                                           ; PI VALUE
CYCLE= (24/FEEDNO)                                          ; FEEDING CYCLE (H)
FEEDWINDOW= 0.4                                             ; DURATION OF FEEDING WINDOW (H)
FIRSTFEEDT= 1                                               ; FIRST FEEDING TIME (H)
MMI1= CYCLE-FEEDWINDOW                                      ; DURATION WHEN THE FUNCTION REMAINS IN STATE 0, REST PERIOD (H)
PSH1= 4 *PI *(MMI1-FIRSTFEEDT)/CYCLE                        ; SINE-WAVE COMPONENT
PER1= 2 *PI *MMI1/CYCLE                                     ; SINE-WAVE COMPONENT
SI1 = SIN ((PI - PER1)/2)                                   ; FIRST SINE-WAVE FUNCTION
SI2 = SIN (2*PI*T / CYCLE+ (PI - PER1+PSH1)/2)              ; SECOND SINE-WAVE FUNCTION
SQW1=(SQRT((SI2-SI1) **2) - (SI2-SI1))/(2*SQRT((SI2-SI1) **2))   ; SQUARE-WAVE FOR TRANSFER FROM BREAST MILK TO INFANT COMPARTMENT
SQW2= (SQW-1) *(-1)                                         ; SQUARE-WAVE FOR TRANSFER FROM PLASMA TO BREAST MILK COMPARTMENT

DADT(1)= - A(1)*K14                                         ; MOTHER-DOSE COMPARTMENT
DADT(2)= A(7)*K72 - A(2)*K23 - A(2)*K28*SQW2 + A(8)*K82*SQW2     ; MOTHER-PQ CENTRAL COMPARTMENT
DADT(3)= A(7)*K73 + A(2)*K23 - A(3)*K30 - A(3)*K39*SQW2 +   ; MOTHER-CPQ CENTRAL COMPARTMENT
A(9)*K93*SQW2
DADT(4)= A(1)*K14 - A(4)*K45                                ; MOTHER-TRANSIT COMPARTMENT 1
DADT(5)= A(4)*K45 - A(5)*K56                                ; MOTHER-TRANSIT COMPARTMENT 2
DADT(6)= A(5)*K56 - A(6)*K67                                ; MOTHER-TRANSIT COMPARTMENT 3
DADT(7)= A(6)*K67 - A(7)*K72 - A(7)*K73                     ; MOTHER-TRANSIT COMPARTMENT 4
DADT(8)= A(2)*K28*SQW2 - A(8)*K82*SQW2 - A(8)*K8T10*SQW1    ; MOTHER-PQ BREASTMILK COMPARTMENT
DADT(9)= A(3)*K39*SQW2 - A(9)*K93*SQW2 - A(9)*K9T13*SQW 1   ; MOTHER-CPQ BREASTMILK COMPARTMENT

DADT(10)= A(8)*K8T10*SQW1 - A(10)*K10T11                    ; INFANT-PQ-DOSE COMPARTMENT
DADT(11)= A(10)*K10T11 - A(11)*K11T12                       ; INFANT-TRANSIT COMPARTMENT 1 FOR PQ
DADT(12)= A(11)*K11T12 - A(12)*K12T16                       ; INFANT-TRANSIT COMPARTMENT 2 FOR PQ

DADT(13)= A(9)*K9T13*SQW1 - A(13)*K13T14                    ; INFANT-CPQ-DOSE COMPARTMENT
DADT(14)= A(13)*K13T14 - A(14)*K14T15                       ; INFANT-TRANSIT COMPARTMENT 1 FOR CPQ
DADT(15)= A(14)*K14T15 - A(15)*K15T17                       ; INFANT-TRANSIT COMPARTMENT 2 FOR CPQ

DADT(16) = A(12)*K12T16 - A(16)*K16T17                      ; INFANT-PQ CENTRAL COMPARTMENT
DADT(17) = A(16)*K16T17 + A(15)*K15T17 - A(17)*K17T0        ; INFANT-CPQ CENTRAL COMPARTMENT

$ERROR
PQPLASMA= (A(2)/S2)                                         ; MOTHER PREDICTED PLASMA PQ
CPQPLASMA= (A(3)/S3)                                        ; MOTHER PREDICTED PLASMA CPQ
PQMILK= (A(8)/S8)                                           ; MOTHER PREDICTED BREASTMILK PQ
CPQMILK= (A(9)/S9)                                          ; MOTHER PREDICTED BREASTMILK CPQ
PQINFANT= (A(16)/S16)                                       ; INFANT PREDICTED PLASMA PQ
CPQINFANT= (A(17)/S17)                                      ; INFANT PREDICTED PLASMA CPQ

IF(CMT.EQ.2.AND.TYPE.EQ.1) THEN                             ;  PREDICTED MOTHER PLASMA PQ WITH ADDITIVE ERROR ON LOG SCALE
    IPRED= A(2)/S2
    W= SQRT (SIGMA (1,1))
    Y= IPRED + EPS (1)
 ENDIF

 IF(CMT.EQ.2.AND.TYPE.EQ.2) THEN                            ;  PREDICTED MOTHER CAPPILLARY PQ WITH ADDITIVE ERROR ON LOG SCALE
    IPRED = (A(2)/S2) *CFPQ
    W     = SQRT (SIGMA (2,2))
    Y = IPRED + EPS (2)
ENDIF

 IF(CMT.EQ.3.AND.TYPE.EQ.1) THEN                            ;  PREDICTED MOTHER PLASMA CPQ WITH ADDITIVE ERROR ON LOG SCALE
    IPRED= A(3)/S3
    W= SQRT (SIGMA (3,3))
    Y= IPRED + EPS (3)
 ENDIF

IF(CMT.EQ.3.AND.TYPE.EQ.2) THEN                             ;  PREDICTED MOTHER CAPPILLARY CPQ WITH ADDITIVE ERROR ON LOG SCALE
    IPRED = (A(3)/S3) *CFCPQ
    W     = SQRT (SIGMA (4,4))
    Y= IPRED + EPS (4)
 ENDIF
```

```
IF(CMT.EQ.4) THEN                                 ;  PREDICTED MOTHER BREAST MILK PQ WITH ADDITIVE ERROR ON LOG SCALE
    IPRED= (A(8)/S8)
    W= SQRT (SIGMA (5,5))
    Y= IPRED + EPS (5)
  ENDIF

  IF(CMT.EQ.5) THEN                               ;  PREDICTED MOTHER BREAST MILK CPQ WITH ADDITIVE ERROR ON LOG SCALE
    IPRED= (A(9)/S9)
    W= SQRT (SIGMA (6,6))
    Y= IPRED + EPS (6)
  ENDIF

IF(IPRED.GT.0) THEN
   IPRED = LOG(IPRED)
  ELSE
   IPRED = 0
  ENDIF

IRES = DV-IPRED
IWRES = IRES/W

$THETA
(1) FIX                                           ; 1. TVF1
(0, 17.1)                                         ; 2. TVCLP
(0, 131)                                          ; 3. TVV2
(0, 0.967)                                        ; 4. TVCLM
(0, 22.7)                                         ; 5. TVV3
(0, 1.44)                                         ; 6. TVMTT
(0, 0.282)                                        ; 7. TVFM
(0, 0.4)                                          ; 8. TVQPQ
(0, 0.376)                                        ; 9. TVPC1_PQ
(0, 0.00889)                                      ; 10. TVPC2_CPQ
(0.898)                                           ; 11. TVCFPQ
(1.06)                                            ; 12. TVCFCPQ

$OMEGA 0.0243                                     ; 1. IIV_F1
$OMEGA 0.0214                                     ; 2. IIV_CLP
$OMEGA 0.0362                                     ; 3. IIV_V2
$OMEGA 0.0688                                     ; 4. IIV_CLM
$OMEGA 0.0307                                     ; 5. IIV_V3
$OMEGA 0.0411                                     ; 6. IIV_MTT
$OMEGA 0.163                                      ; 7. IIV_FM
$OMEGA 0.590                                      ; 8. IIV_QPRQ
$OMEGA 0 FIX                                      ; 9. IIV_PC1
$OMEGA 0 FIX                                      ; 10. IIV_PC2
$OMEGA BLOCK (1) 0.0413                           ; 11. IOV_F1_OCC1
$OMEGA BLOCK (1) SAME                             ; 12. IOV_F1_OCC2
$OMEGA BLOCK (1) SAME                             ; 13. IOV_F1_OCC3
$OMEGA BLOCK (1) SAME                             ; 14. IOV_F1_OCC4
$OMEGA BLOCK (1) 0.280                            ; 15. IOV_MTT_OCC1
$OMEGA BLOCK (1) SAME                             ; 16. IOV_MTT_OCC2
$OMEGA BLOCK (1) SAME                             ; 17. IOV_MTT_OCC3
$OMEGA BLOCK (1) SAME                             ; 18. IOV_MTT_OCC4

$SIGMA
0.102                                             ; 1. ADDITIVE ERROR FOR PQ IN PLASMA
0.057                                             ; 2. ADDITIVE ERROR FOR PQ IN CAPILLARY
0.0198                                            ; 3. ADDITIVE ERROR FOR CPQ IN PLASMA
0.0115                                            ; 4. ADDITIVE ERROR FOR CPQ IN CAPILLARY
0.156                                             ; 5. ADDITIVE ERROR FOR PQ IN BREASTMILK
0.0911                                            ; 6. ADDITIVE ERROR FOR CPQ IN BREASTMILK

$SIM (123456) ONLYSIM SUBPROBLEMS=1000
```

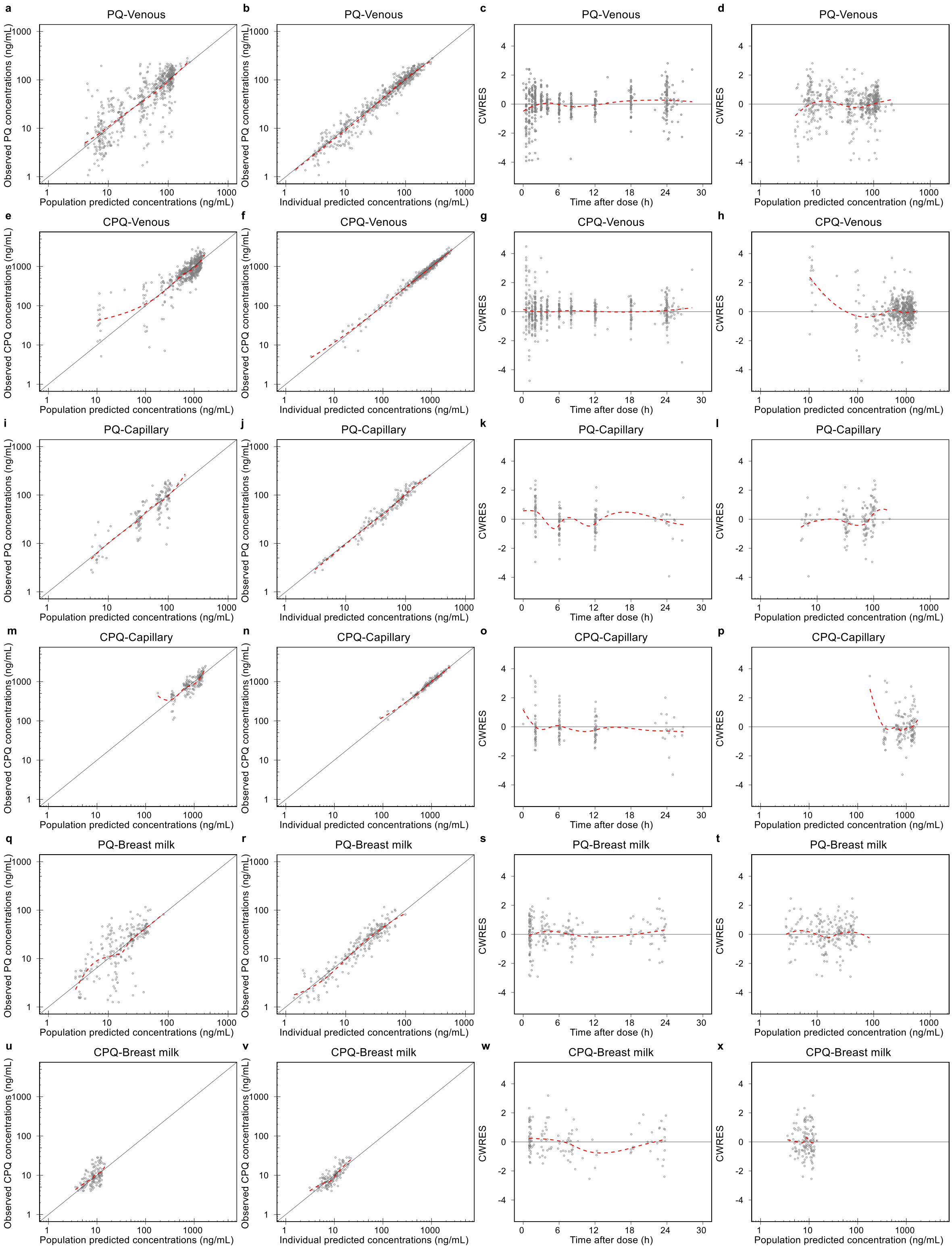

Supplementary Figure 1. Goodness of fits from the final population model of primaquine (PQ) and carboxyprimaquine (CPQ) in breastfeeding women in each sample matrix. **a, e, i, m, q, u** observed concentrations vs population predictions. **b, f, j, n r, v** observed concentrations vs individually predicted concentrations. **c, g, k, o, s, w** conditionally weighted residuals (CWRES) vs time after dose. **d, h, l, p, t, x** conditionally weighted residuals vs population predictions. The open circles represent the observed concentrations. The solid black lines represent the line of identity or zero-line and the dashed red lines represent a local polynomial regression fitting of all data (trend lines).

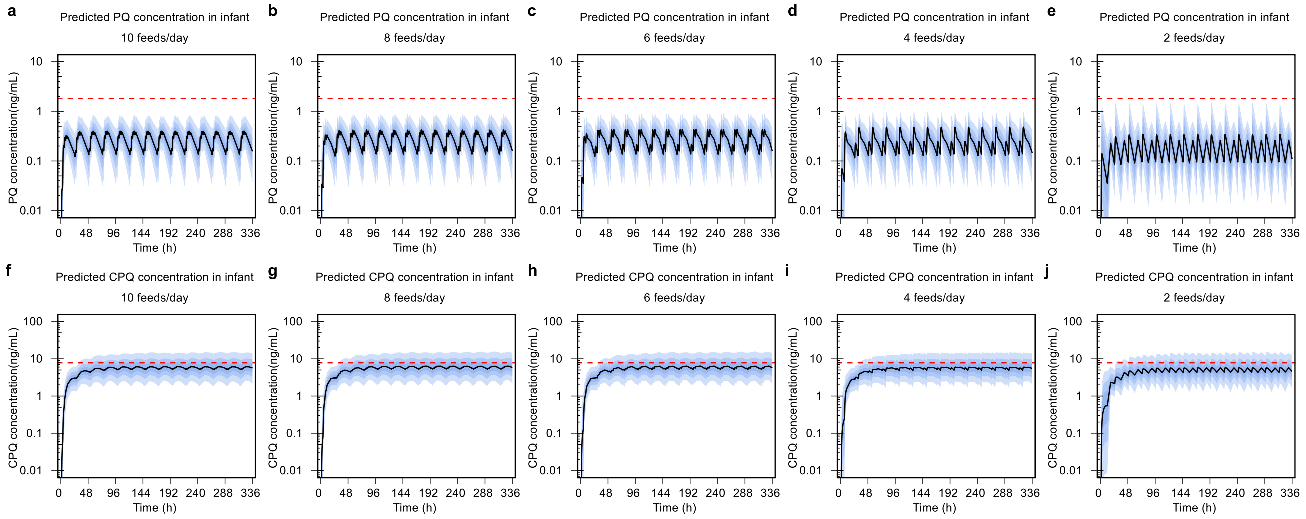

Supplementary Figure 2. Simulation results from the sensitivity analysis of breastfeeding pattern using a standard dose of primaquine (0.5 mg base/kg once daily for 14 days) in the mothers (n=1,000). a Predicted primaquine concentration in infants receiving 10 breastfeeds/day. **b** Predicted primaquine concentration in infants receiving 8 breastfeeds/day. **c** Predicted primaquine concentration in infants receiving 6 breastfeeds/day. **d** Predicted primaquine concentration in infants receiving 4 breastfeeds/day. **e** Predicted primaquine concentration in infants receiving 2 breastfeeds/day. **f** Predicted carboxyprimaquine concentration in infants receiving 10 breastfeeds/day. **g** Predicted carboxyprimaquine concentration in infants receiving 8 breastfeeds/day. **h** Predicted carboxyprimaquine concentration in infants receiving 6 breastfeeds/day. **i** Predicted carboxyprimaquine concentration in infants receiving 4 breastfeeds/day. **j** Predicted carboxyprimaquine concentration in infants receiving 2 breastfeeds/day. The solid black lines represent the median of the simulations. The shaded areas represent the 95% prediction intervals of the simulations. The dashed red lines represent the median lower limit of quantification of primaquine (1.82 ng/mL) and carboxyprimaquine (7.81 ng/mL) reported in capillary samples. PQ; primaquine, CPQ; carboxyprimaquine

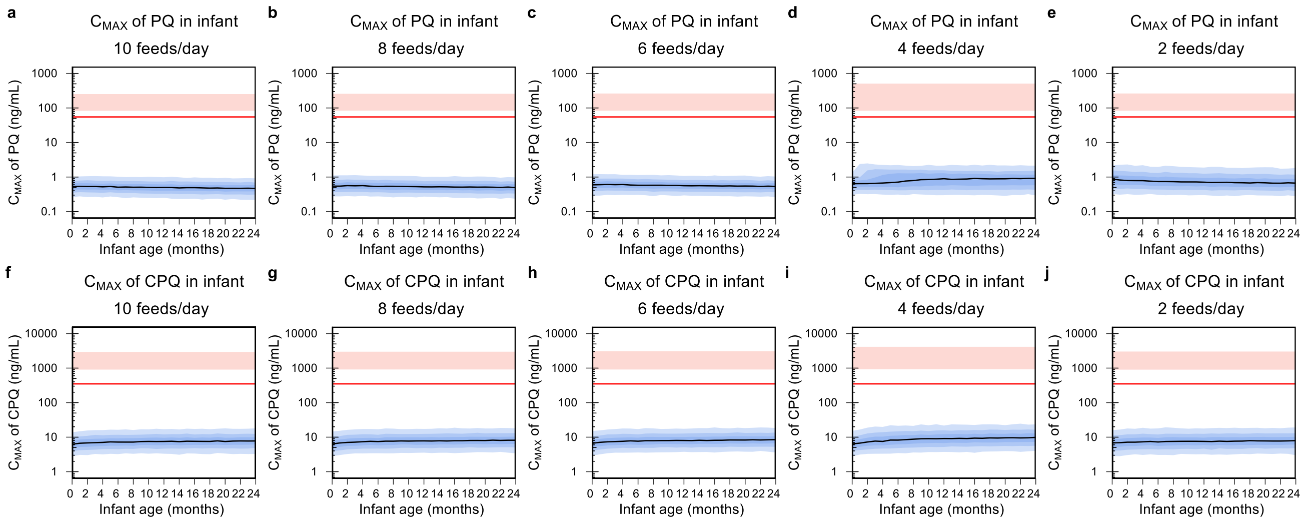

Supplementary Figure 3. Simulated C_{MAX} from the sensitivity analysis of breastfeeding pattern using a standard dose of primaquine (0.5 mg base/kg once daily for 14 days) in the mothers (n=1,000). **a** Predicted primaquine C_{MAX} in infants receiving 10 breastfeeds/day. **b** Predicted primaquine C_{MAX} in infants receiving 8 breastfeeds/day. **c** Predicted primaquine C_{MAX} in infants receiving 6 breastfeeds/day. **d** Predicted primaquine C_{MAX} in infants receiving 4 breastfeeds/day. **e** Predicted primaquine C_{MAX} in infants receiving 2 breastfeeds/day. **f** Predicted carboxyprimaquine C_{MAX} in infants receiving 10 breastfeeds/day. **g** Predicted carboxyprimaquine C_{MAX} in infants receiving 8 breastfeeds/day. **h** Predicted carboxyprimaquine C_{MAX} in infants receiving 6 breastfeeds/day. **i** Predicted carboxyprimaquine C_{MAX} in infants receiving 4 breastfeeds/day. **j** Predicted carboxyprimaquine C_{MAX} in infants receiving 2 breastfeeds/day. The solid black lines represent the median of the simulations in infants. The blue shaded areas represent the 95% prediction intervals of the simulations in infants. The red shaded areas represent the 95% prediction interval of the simulated C_{MAX} in the mothers with 60 kg body weight. The red solid lines represent the median C_{MAX} (Primaquine (PQ): 55.0 ng/mL, Carboxyprimaquine (CPQ): 349 ng/mL) of the mother receiving single low dose of primaquine (0.25 mg base/kg), a conservative reference for primaquine and carboxyprimaquine concentration known not to cause significant haemolysis in G6PD deficient individuals.

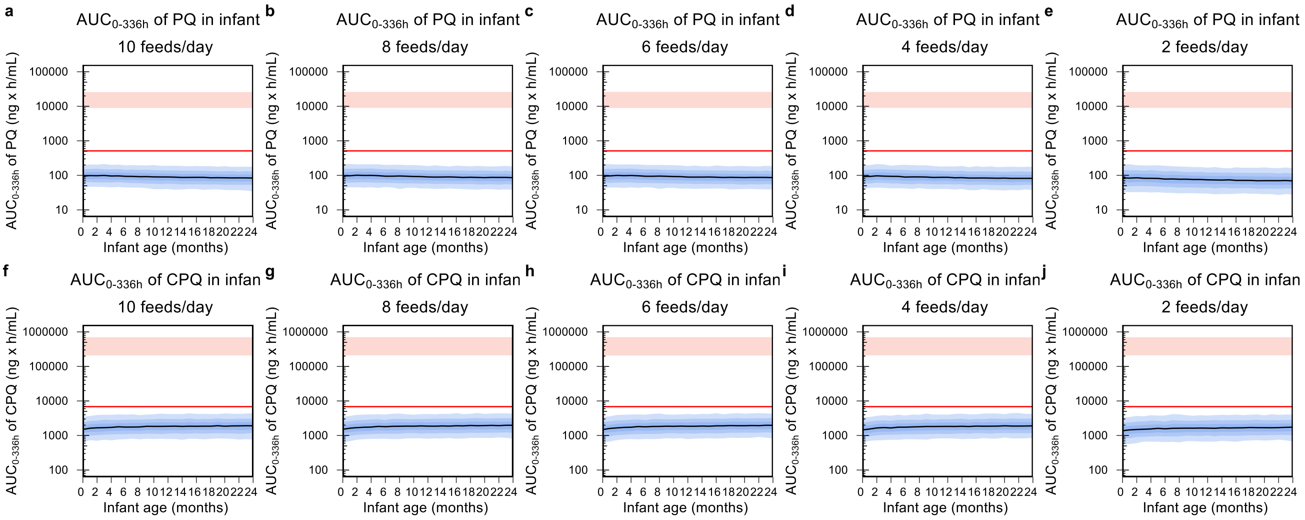

Supplementary Figure 4. Simulated $AUC_{0\text{-}336h}$ from the sensitivity analysis of breastfeeding pattern using a standard dose of primaquine (0.5 mg base/kg once daily for 14 days) in the mothers (n=1,000). a Predicted primaquine $AUC_{0\text{-}336h}$ in infants receiving 10 breastfeeds/day. **b** Predicted primaquine $AUC_{0\text{-}336h}$ in infants receiving 8 breastfeeds/day. **c** Predicted primaquine $AUC_{0\text{-}336h}$ in infants receiving 6 breastfeeds/day. **d** Predicted primaquine $AUC_{0\text{-}336h}$ in infants receiving 4 breastfeeds/day. **e** Predicted primaquine $AUC_{0\text{-}336h}$ in infants receiving 2 breastfeeds/day. **f** Predicted carboxyprimaquine $AUC_{0\text{-}336h}$ in infants receiving 10 breastfeeds/day. **g** Predicted carboxyprimaquine $AUC_{0\text{-}336h}$ in infants receiving 8 breastfeeds/day. **h** Predicted carboxyprimaquine $AUC_{0\text{-}336h}$ in infants receiving 6 breastfeeds/day. **i** Predicted carboxyprimaquine $AUC_{0\text{-}336h}$ in infants receiving 4 breastfeeds/day. **j** Predicted carboxyprimaquine $AUC_{0\text{-}336h}$ in infants receiving 2 breastfeeds/day. The solid black lines represent the median of the simulations in infants. The blue shaded areas represent the 95% prediction intervals of the simulations in infants. The red shaded areas represent the 95% prediction interval of the simulated $AUC_{0\text{-}336h}$ in the mothers with 60 kg body weight. The red solid lines represent the median $AUC_{0\text{-}24h}$ (Primaquine (PQ): 512 ng × h/mL, Carboxyprimaquine (CPQ): 6848 ng × h/mL) of the mother receiving single low dose of primaquine (0.25 mg base/kg), a conservative reference for primaquine and carboxyprimaquine concentration known not to cause significant haemolysis in G6PD deficient individuals.

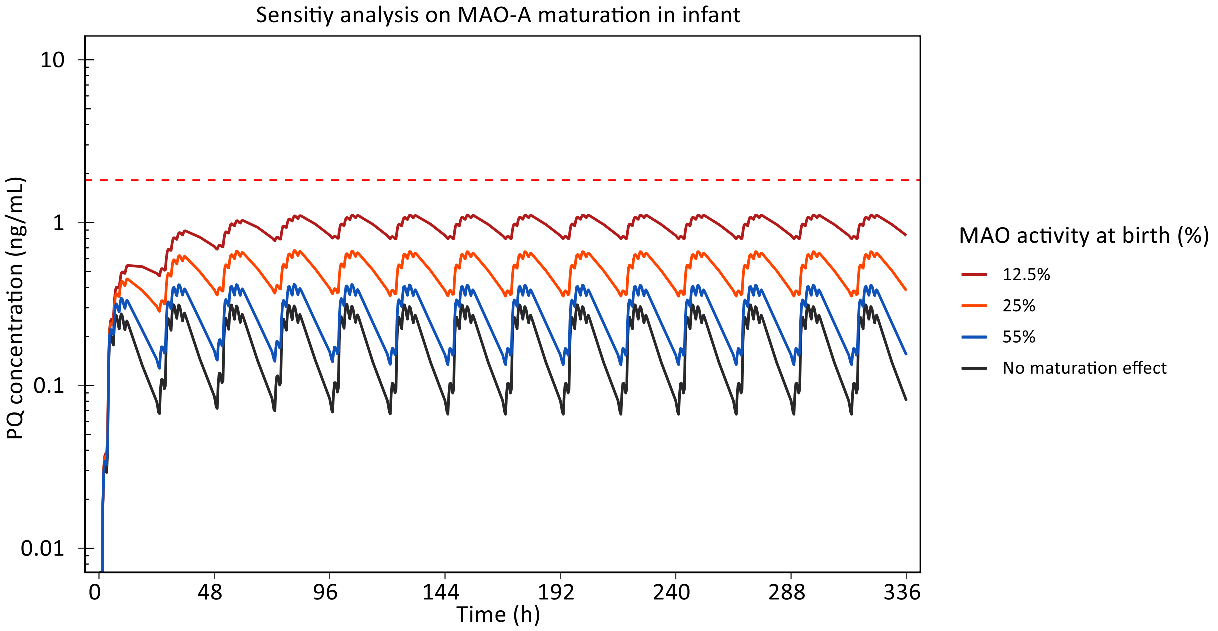

Supplementary Figure 5. Simulated median primaquine concentration in infants with varying MAO-A enzyme activity at full-term birth (n=1,000). The horizontal dashed red line represents the lower limit of quantification of primaquine reported in infants (1.82 ng/mL)

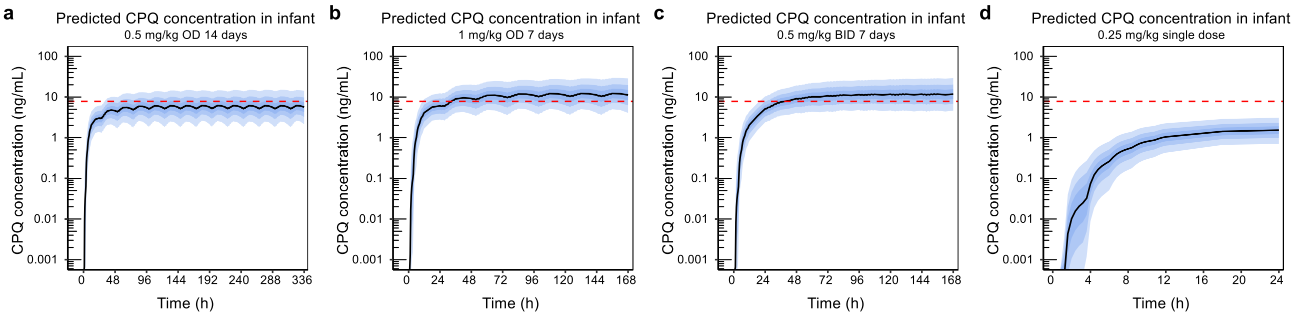

Supplementary Figure 6. Simulated concentration-time profile of carboxyprimaquine (CPQ) from the mother-to-infant model using different dosing regimens (n=1,000). a 0.5 mg/kg once daily for 14 days. **b** 1 mg/kg once daily for 7 days. **c** 0.5 mg/kg twice daily for 7 days. **d** 0.25 mg/kg single dose. The solid black lines represent the median of the simulations. The shaded areas represent the 95% prediction intervals of the simulations. The dashed red lines represent the median lower limit of quantification of carboxyprimaquine (CPQ, 7.81 ng/mL) reported in infant capillary samples.

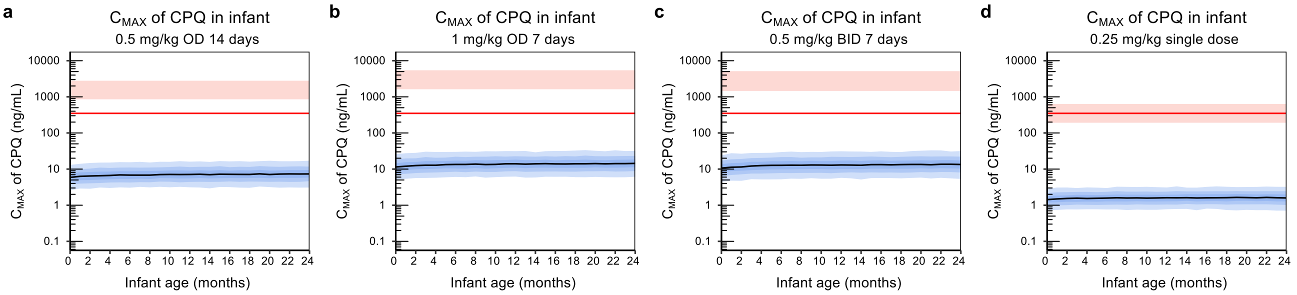

Supplementary Figure 7. Simulated C_{MAX} of carboxyprimaquine (CPQ) from the mother-to-infant model using different dosing regimens (n=1,000). a 0.5 mg/kg once daily for 14 days. **b** 1 mg/kg once daily for 7 days. **c** 0.5 mg/kg twice daily for 7 days. **d** 0.25 mg/kg single dose. The solid black lines represent the median of the simulations in infants. The blue shaded areas represent the 95% prediction intervals of the simulations in infants. The red shaded areas represent the 95% prediction interval of the simulated C_{MAX} in the mothers with 60 kg body weight of each dosing scenario. The red solid lines represent the median C_{MAX} (349 ng/mL) of the mother receiving single low dose of primaquine (0.25 mg/kg), a conservative reference for carboxyprimaquine concentration known not to cause significant haemolysis in G6PD deficient individuals.

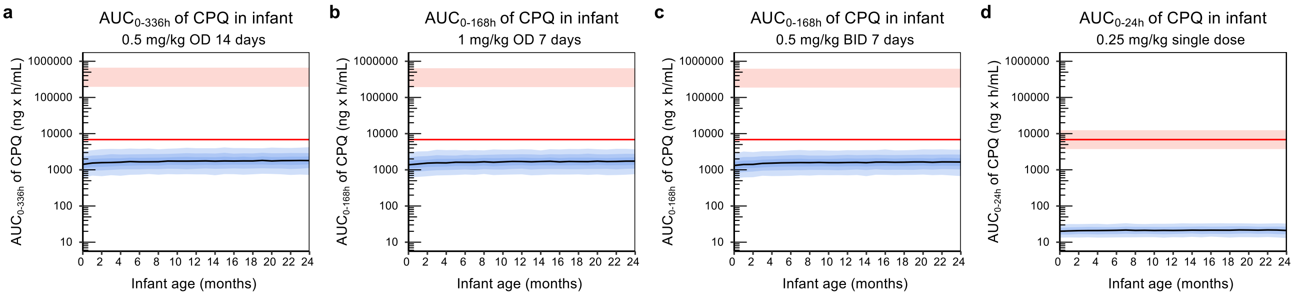

Supplementary Figure 8. Simulated AUC of carboxyprimaquine (CPQ) from the mother-to-infant model using different dosing regimens (n=1,000). a 0.5 mg/kg once daily for 14 days. **b** 1 mg/kg once daily for 7 days. **c** 0.5 mg/kg twice daily for 7 days. **d** 0.25 mg/kg single dose. The solid black lines represent the median of the simulations in infants. The blue shaded areas represent the 95% prediction intervals of the simulations in infants. The red shaded areas represent the 95% prediction interval of the simulated AUC in the mothers with 60 kg body weight. The red solid lines represent the median $AUC_{0\text{-}24h}$ (6848 ng × h/mL) of the mother receiving single low dose of primaquine (0.25 mg/kg), a reference for a carboxyprimaquine exposure known not to cause significant haemolysis in G6PD deficient individuals.

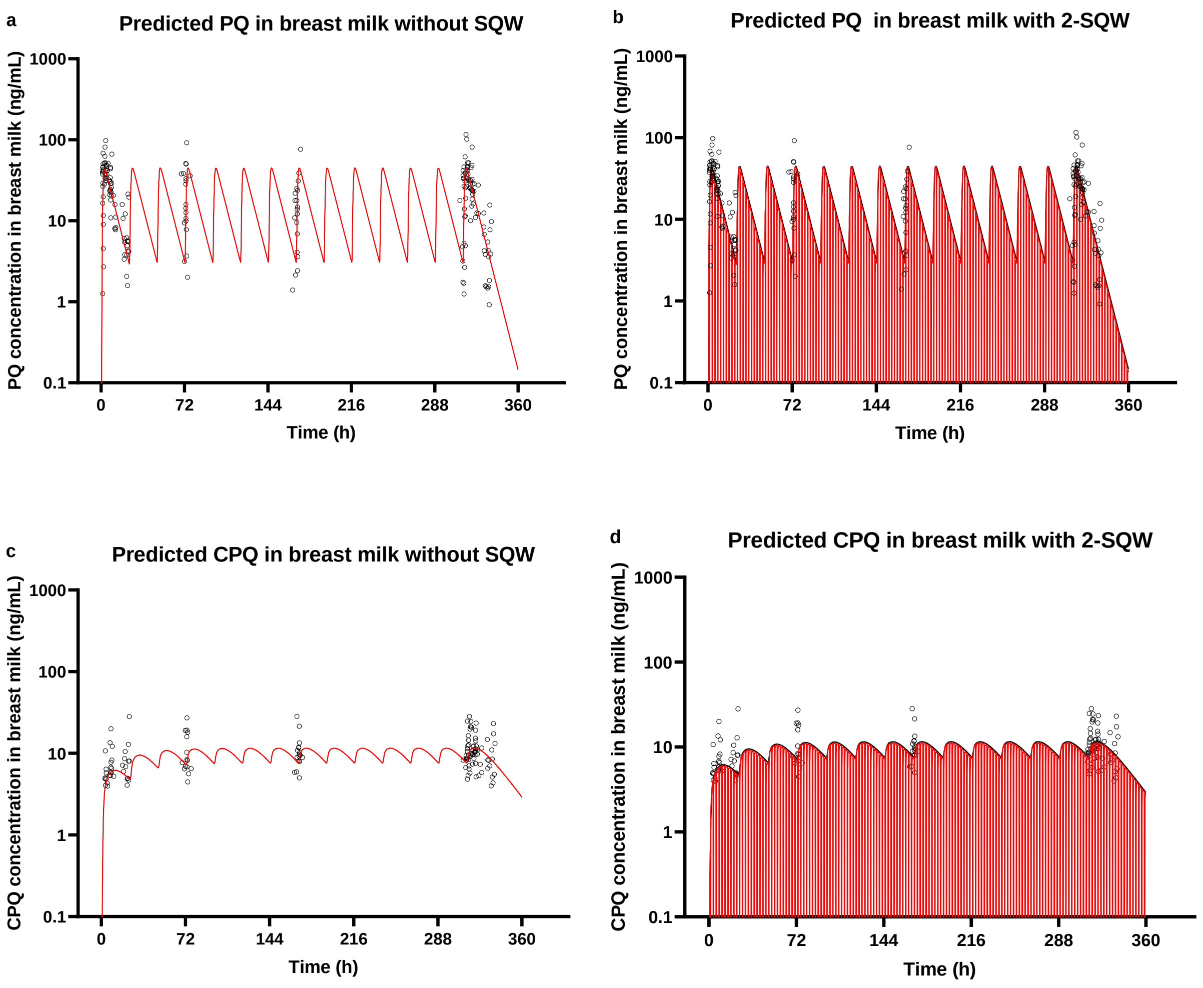

Supplementary Figure 9. Simulated mean breast milk concentrations using a model with and without square-wave functions. The simulated mean breast milk concentrations overlaid with observed breast milk concentrations. **a** Predicted primaquine concentration in breast milk from a model without square-wave function. **b** Predicted primaquine concentration in breast milk from a model with square-wave functions. **c** Predicted carboxyprimaquine concentration in breast milk from a model without square-wave function. **d** Predicted carboxyprimaquine concentration in breast milk from a model with square-wave functions. PQ; primaquine, CPQ; carboxyprimaquine, SQW; square-wave function.
