# Supplementary material for: Population pharmacokinetic modelling of primaquine exposures in lactating women and breastfed infants
Source: Nat Commun. 2024 May 8;15:3851. doi: 10.1038/s41467-024-47908-y (PMC11078975; doi:10.1038/s41467-024-47908-y)
Supplement: Supplementary file 3 — Reporting Summary [file 41467_2024_47908_MOESM3_ESM.pdf]

nature portfolio

Corresponding author(s): Richard M. Hoglund

Last updated by author(s): Apr 5, 2024

# Reporting Summary

Nature Portfolio wishes to improve the reproducibility of the work that we publish. This form provides structure for consistency and transparency in reporting. For further information on Nature Portfolio policies, see our Editorial Policies and the Editorial Policy Checklist.

## Statistics

For all statistical analyses, confirm that the following items are present in the figure legend, table legend, main text, or Methods section.

| n/a | Confirmed | |
|---|---|---|
| ☐ | ☒ | The exact sample size (*n*) for each experimental group/condition, given as a discrete number and unit of measurement |
| ☐ | ☒ | A statement on whether measurements were taken from distinct samples or whether the same sample was measured repeatedly |
| ☐ | ☒ | The statistical test(s) used AND whether they are one- or two-sided<br>*Only common tests should be described solely by name; describe more complex techniques in the Methods section.* |
| ☐ | ☒ | A description of all covariates tested |
| ☐ | ☒ | A description of any assumptions or corrections, such as tests of normality and adjustment for multiple comparisons |
| ☐ | ☒ | A full description of the statistical parameters including central tendency (e.g. means) or other basic estimates (e.g. regression coefficient) AND variation (e.g. standard deviation) or associated estimates of uncertainty (e.g. confidence intervals) |
| ☐ | ☒ | For null hypothesis testing, the test statistic (e.g. *F*, *t*, *r*) with confidence intervals, effect sizes, degrees of freedom and *P* value noted<br>*Give P values as exact values whenever suitable.* |
| ☒ | ☐ | For Bayesian analysis, information on the choice of priors and Markov chain Monte Carlo settings |
| ☒ | ☐ | For hierarchical and complex designs, identification of the appropriate level for tests and full reporting of outcomes |
| ☒ | ☐ | Estimates of effect sizes (e.g. Cohen's *d*, Pearson's *r*), indicating how they were calculated |

*Our web collection on statistics for biologists contains articles on many of the points above.*

## Software and code

Policy information about availability of computer code

| | |
|---|---|
| Data collection | No software was used for data collection. |
| Data analysis | Data analysis was performed in NONMEM version 7.4 (Icon Development Solution, Ellicott City, MD). Automations, model evaluations, model diagnostics and model support were carried out using the R-package Xpose version 4.0, Perl-speaks-NONMEM version 4.8.0, as well as Pirana version 2.9.9. NONMEM code described both mother and mother-to-infant model used in this study was provided in the supplementary materials and NONMEM model file was also deposited to Zenodo repository (https://doi.org/10.5281/zenodo.10925291). |

For manuscripts utilizing custom algorithms or software that are central to the research but not yet described in published literature, software must be made available to editors and reviewers. We strongly encourage code deposition in a community repository (e.g. GitHub). See the Nature Portfolio guidelines for submitting code & software for further information.

## Data

Policy information about availability of data

All manuscripts must include a data availability statement. This statement should provide the following information, where applicable:

- Accession codes, unique identifiers, or web links for publicly available datasets
- A description of any restrictions on data availability
- For clinical datasets or third party data, please ensure that the statement adheres to our policy

The patient data are available under restricted access for ethical reason, access can be obtained by written application to the Data Access Committee at Mahidol Oxford Tropical Medicine Research Unit (datasharing@tropmedres.ac). Applications are commonly reviewed within 2 weeks.

## Research involving human participants, their data, or biological material

Policy information about studies with human participants or human data. See also policy information about sex, gender (identity/presentation), and sexual orientation and race, ethnicity and racism.

| | |
|---|---|
| Reporting on sex and gender | The pharmacokinetic properties of primaquine in lactating women only apply to one sex, i.e., women. In the case of infants, the sex of the infants was collected in the clinical study. However, we did not use this information in the current analysis. |
| Reporting on race, ethnicity, or other socially relevant groupings | This study took place at 3 of the Shoklo Malaria Research Unit (SMRU) clinics along the Thailand–Myanmar border, which serve migrant workers and refugees from Myanmar, predominantly from the Burman or Karen ethnic groups. |
| Population characteristics | The study enrolled women aged ≥18 years (range: 18-40) with a history of P. vivax infection and no prior radical cure by primaquine treatment, who were breastfeeding healthy infants. |
| Recruitment | Women aged ≥18 years with a history of P. vivax infection, no prior radical cure with primaquine treatment, and who were breastfeeding healthy infants were invited to enroll in the study. Eligible mothers received counseling from trained staff, ensuring standard procedures for informed consent, considering preferred language and literacy. Women had the option to make their decision at a subsequent visit if necessary. Consenting mothers and their infants underwent both complete blood count and G6PD testing. Any detected abnormalities in either the mother or the infant resulted in their exclusion from the study. |
| Ethics oversight | The clinical study was approved by 3 ethical review bodies: the Tak Community Advisory Board; the Ethics Committee of the Faculty of Tropical Medicine, Mahidol University in Bangkok (TMEC 12–036); and the Oxford Tropical Research Ethic Committee (OXTREC 28-12). |

Note that full information on the approval of the study protocol must also be provided in the manuscript.

# Field-specific reporting

Please select the one below that is the best fit for your research. If you are not sure, read the appropriate sections before making your selection.

☒ Life sciences ☐ Behavioural & social sciences ☐ Ecological, evolutionary & environmental sciences

For a reference copy of the document with all sections, see nature.com/documents/nr-reporting-summary-flat.pdf

# Life sciences study design

All studies must disclose on these points even when the disclosure is negative.

| | |
|---|---|
| Sample size | No formal sample size calculation was performed. The number of patients was limited to 21 pairs (mother/child) as this was a study to gather information about primaquine in lactation through detailed pharmacokinetics sampling. The number was chosen to give adequate data to describe the basic pharmacokinetics of primaquine in this group without involving an unnecessarily large numbers of infants in the detailed sampling. |
| Data exclusions | No data were excluded from the analysis. |
| Replication | The internal model validation performed in this study, i.e., simulation-based diagnostics (visual predictive checks), showed that the model was reproducible. |
| Randomization | This pharmacokinetic study was a descriptive study conducted in lactating women and their infants. Randomization was not relevant to this study as the study was designed to evaluate the pharmacokinetic properties of a single drug administered i.e. primaquine in a specific population i.e. lactating women and their infants. Thus, there is no comparator in this study. |
| Blinding | Blinding was not relevant to this descriptive study, as all lactating women received the study drug. |

# Reporting for specific materials, systems and methods

We require information from authors about some types of materials, experimental systems and methods used in many studies. Here, indicate whether each material, system or method listed is relevant to your study. If you are not sure if a list item applies to your research, read the appropriate section before selecting a response.

## Materials & experimental systems

| n/a | Involved in the study |
|---|---|
| ☒ | ☐ Antibodies |
| ☒ | ☐ Eukaryotic cell lines |
| ☒ | ☐ Palaeontology and archaeology |
| ☒ | ☐ Animals and other organisms |
| ☐ | ☒ Clinical data |
| ☒ | ☐ Dual use research of concern |
| ☒ | ☐ Plants |

## Methods

| n/a | Involved in the study |
|---|---|
| ☒ | ☐ ChIP-seq |
| ☒ | ☐ Flow cytometry |
| ☒ | ☐ MRI-based neuroimaging |

## Clinical data

Policy information about clinical studies

All manuscripts should comply with the ICMJE guidelines for publication of clinical research and a completed CONSORT checklist must be included with all submissions.

| | |
|---|---|
| Clinical trial registration | NCT01780753 |
| Study protocol | The protocol is available from the authors upon reasonable request. |
| Data collection | The data in this analysis were obtained from a clinical trial conducted at three clinics at Shoklo Malaria Research Unit. The clinics were located at the Thai-Myanmar border and the study was conducted between 11 November 2012 and 24 June 2014. |
| Outcomes | The primary outcomes were the pharmacokinetic parameters of primaquine and carboxyprimaquine in the breast milk of lactating women treated with primaquine for the radical treatment of P. vivax, assessed by measuring primaquine and carboxyprimaquine concentrations in breast milk and conducting population pharmacokinetic analysis. The secondary outcomes included primaquine and carboxyprimaquine pharmacokinetic parameters in the blood of lactating women and their breastfed infants, assessed by measuring primaquine and carboxyprimaquine concentrations in blood and conducting population pharmacokinetic analysis. |

## Plants

| | |
|---|---|
| Seed stocks | n/a |
| Novel plant genotypes | n/a |
| Authentication | n/a |
